# Integrin subunits alpha5 and alpha6 regulate cell cycle by modulating the chk1 and Rb/E2F pathways to affect breast cancer metastasis

**DOI:** 10.1186/1476-4598-10-84

**Published:** 2011-07-13

**Authors:** Yanfang Wang, Sylvia Shenouda, Somesh Baranwal, Rajamani Rathinam, Prachi Jain, Lili Bao, Siddhartha Hazari, Srikanta Dash, Suresh K Alahari

**Affiliations:** 1 Department of Biochemistry and Molecular Biology, Stanley Scott Cancer Center, LSU Health Sciences Center, New Orleans, LA 70112, USA; 2 Department of Pathology and Laboratory Medicine, Tulane University Medical School, New Orleans, LA 70112, USA

## Abstract

**Background:**

Although integrins have been implicated in the progression of breast cancer, the exact mechanism whereby they exert this regulation is clearly not understood. To understand the role of integrins in breast cancer, we examined the expression levels of several integrins in mouse breast cancer cell lines by flow cytometry and the data were validated by Western and RT-PCR analysis. The importance of integrins in cell migration and cell invasion was examined by *in vitro* assays. Further the effect of integrins on metastasis was investigated by *in vivo *experimental metastasis assays using mouse models.

**Results:**

Integrin α5 subunit is highly expressed in the nonmetastatic cell line 67NR and is significantly low in the highly invasive cell line 4T1. In contrast, expression levels of integrin α6 subunit are high in 4T1 cells and low in 67NR cells. *In vitro *data indicated that overexpression of α5 subunit and knockdown of α6 integrin subunit inhibited cell proliferation, migration, and invasion. Our *in vivo *findings indicated that overexpression of integrin α5 subunit and knockdown of α6 subunit decreased the pulmonary metastasis property of 4T1 cells. Our data also indicated that overexpression of alpha 5 integrin subunit and suppression of alpha6 integrin subunit inhibited cells entering into S phase by up-regulating p27, which results in downregulation of cyclinE/CDK2 complexes, This suggests that these integrins regulate cell growth through their effects on cell-cycle-regulated proteins. We also found that modulation of these integrins upregulates E2F, which may induce the expression of chk1 to regulate cdc25A/cyclin E/CDK2/Rb in a feedback loop mechanism.

**Conclusion:**

This study indicates that Integrin α5 subunit functions as a potential metastasis suppressor, while α6 subunit functions as a metastasis promoter. The modulation of integrins reduces cdc25 A, another possible mechanism for downregulation of CDK2. Taken together we demonstrate a link between integrins and the chk1-cdc25-cyclin E/CDK2-Rb pathway.

## Background

Tumor metastasis is a highly complex multistep process involving unregulated cell growth, cell-cell and cell-matrix interactions, cell adhesion, angiogenesis, and growth of new cancer colonies [1]. During these steps, the expression level of some genes is altered. Thus, these genes, once identified, can serve as bio-markers of metastatic diagnosis and prognosis. Over the past decade, a variety of genes have been identified that are altered in tumor growth and metastasis [2].

Integrins are a family of transmembrane glycoprotein adhesion receptors that mediate cell-matrix and cell-cell adhesion. They are the main receptors for sensing the extracellular environment of the cell [3]. Integrins form heterodimers of α and β subunits [4]. Eighteen α subunits and eight β subunits can associate to form 24 unique integrin heterodimers. Numerous studies have shown that in addition to sensing the extracellular environment, integrins are involved in various intracellular pathways, including cell adhesion, migration, polarity, survival, growth and death [3,5,6], suggesting their important role in cancer [4]. Furthermore, integrins were shown to be differentially expressed during tumor growth and progression, making them potential targets for cancer diagnosis and therapy [3,5,6].

The mechanisms whereby integrins function in tumor cells are yet to be determined. However, some cancer-related proteins, such as focal adhesion kinase (FAK) [7] and Nischarin [8] can bind to and interact with integrins. Therefore, we investigated whether integrins are differentially expressed in highly metastatic cells compared with non-metastatic cells, and examined the effect of these differentially expressed integrins on cell proliferation, invasion and the potential of metastasis.

In this study we used mouse breast cancer cell lines 4T1, 4T07, and 67NR because they represent a good model for the study of breast cancer metastasis. 4T1, 4T07, and 67NR are derived from the same BALB/c mammary tumor and are highly tumorigenic, but vary in their metastatic potential. 4T1 widely disseminates, resulting in secondary tumors in the lung, liver, bone, and brain; 4T07 spreads to the lung and liver but cannot establish metastatic nodules; 67NR does not metastasize [9,10]. Our study shows that integrin α5 subunit functions as a candidate metastasis suppressor, while α6 subunit promotes tumor metastasis in 4T1 cell lines through the modulation of pathways regulated by the cell cycle.

## Materials and methods

### Cell lines

Mouse mammary carcinoma cells 4T1, 4T07, and 67NR were cultured in Dulbecco's Modified Eagle's Medium (DMEM) supplemented with 10% fetal bovine serum (FBS). Cells were incubated at 37°C with 5% (w/v) CO_2 _and 95% (w/v) air mixture.

### Flow cytometry

One million 4T1, 4T07, and 67NR cells were incubated with primary antibodies against integrins α4 (clone PS/2), α5 (clone 5H10-27), α6 (clone NKI-GoH3), β1 (clone MB1.2), β2 (clone M18/2), α2β1 (clone BMA2.1), α5β1 (clone BMA5), and αvβ6 (clone 10D5) for 30 min at 4°C in phosphate-buffered saline containing 1% FBS. Cells were then washed twice with ice-cold PBS and incubated with FITC-conjugated secondary antibodies (1:1,250) for 30 min at 4°C. Most antibodies were purchased from Millipore (Madison, WI); the exception was integrin α5, which was purchased from Abcam (Cambridge, MA). Relative amounts of cell surface integrins were determined using a FACScan cytometer with CellQuest software (Becton-Dickinson, San Jose, CA).

### RT-PCR

Total RNAs from these cell lines were isolated using a RNeasy kit (Qiagen, Valencia, CA). RNA quality was determined with the Agilent Bioanalyser 2100 and RNA Nano 6000 Labchip kit (Agilent Technologies, Palo Alto, CA). RNA concentrations were determined with a NanoDrop apparatus (NanoDrop Technologies). RT-PCR was done to confirm expression of integrins α5 and α6 by one-step RT-PCR (Qiagen). Briefly, the 25 μl RT-PCR reaction included 100 ng total RNA, 5 μl 5 × RT-PCR buffer, 0.4 mM dNTP, 0.6 μM forward and reverse primers, and 1 μl enzyme mix. The reactions were incubated in a 96-well plate at 50°C for 30 min and 95°C for 15 min, followed by 25 cycles of 95°C for 15 sec, 60°C for 30 sec, 72°C for 30 s, and 72°C for 7 min. The primers used in RT-PCR were:

For integrin α5: forward: 5'-GGACCAAGACGGCTACAATGATGT-3';

Reverse: 5'-ACCTGGGAAGGTTTAGTGCTCAGT-3'.

For Integrin α6: forward: 5'-AAAGAGACATGAAGTCCGCGCATC-3';

Reverse: 5'-AACAATGTCTTGCCACCCATCTGC-3'.

For CHK1: forward: 5'-TGGACAAACTGGTTCAGGGCAT-3';

Reverse: 5'-TGCTCACAACATCGCTGAGCTT-3'.

For E2F1: forward: 5'-ACCCAGGGAAAGGTGTGAAA-3';

Reverse: 5'-AAAGCAGTTGCAGCTGTGTGGT-3'.

For GAPDH forward: 5'-AACATCATCCCTGCATCCACTGGT-3';

Reverse: 5'-TGTTGAAGTCGCAGGAGACAACCT-3'.

The RT-PCR products were determined by electrophoresis on 2% agarose gel stained with ethidium bromide.

### Western blotting

Cells were washed twice using ice cold PBS. Cell lysates were prepared using RIPA buffer (50 mM Tris, pH 7.5; 1% NP-40; 0.1% sodium deoxycholate; 150 mM NaCl; 50 mM NaF; and 1 mM sodium pyrophosphate). Lysates were centrifuged at 20,000 × *g *for 20 min at 4°C. Small aliquots (10 μl) of the supernatant were used for determining protein concentrations by a bicinchonic acid assay (Pierce; Rockford, IL). Proteins (20-50 μg) were separated by SDS-polyacrylamide electrophoresis on 6% or 12% polyacrylamide gels and transferred to PVDF membranes (GE Healthcare Biosciences, Piscataway, NJ). Blots were blocked in 5% milk and 0.1% Tris-buffered saline-Tween 20 for 1 hr at room temperature. Blots were incubated with antibodies against integrin α5 subunit (1:1000; Millipore, Madison, WI) and integrin α6 subunit (1:500; Santa Cruz Biotechnology, Santa Cruz, CA). Membranes were washed 4X for 5 min each time using PBS Tween 20 and incubated with anti-mouse or anti-rabbit horseradish-peroxidase-conjugated secondary antibodies (1:2,000; Santa Cruz Biotechnology). Detection of the signal was done using an ECL immunoblotting detection system (GE Healthcare Biosciences).

### Construction of stable cell lines overexpressing integrin α5 and knockdown integrin α6

We used the expression plasmid pcDNA 3.1/ITGA5, which contains the full length of integrin α5 subunit, to transfect 4T1 cells by the Lipofectamine 2000 method (Life Technologies, Rockville, MD). The transfected cells were subjected to 1 mg/mL G418 selection. Several G418-resistant clones were tested for integrin α5 subunit overexpression. The mixture of positive clones was used for further studies.

Two integrin α6 subunit oligos were designed by selecting appropriate sequences from the mouse integrin α6 gene (NM_008397) and named shRNA-1 and shRNA-control. The sequences of the two shRNAs were as follows:

shRNA-: 5'-GATCCCCCCTTGTACACGGATTGAATTTCAAGAGAATT CAATCCGTGTACAAGGTTTTTA-3';

shRNA-control: 5'-GATCCCCGGACAACGTGATCCGGAA ATTCAAGAGATTTCCGGATCACGTTGTCCTTTTTA-3'

The double strands for shRNA-1 and shRNA-control oligos were cloned into GeneScript shRNA expression vector, pRNAT-H1.1/Neo (Cat# SD1213-Genescript, NJ), using the BamH1 and HindIII restriction enzyme sites. The oligo insertion was confirmed by sequencing. After plasmid transfection, cells were cultured in G-418 (500 ug/ml) and several clones were isolated. The individual clones were characterized and positive clones were selected RT-PCR and Western blot analysis.

### Cell proliferation analysis

To examine cell proliferation, we used MTT calorimetry assays (Millipore, Madison, WI). Cells were harvested, resuspended, and plated in 96-well plates at 1 × 10^3 ^cells per well in 100 μL of cell culture medium and maintained at 37°C in a humidified incubator containing 5% CO2. After every 24 h, 10 μL of the MTT solution was added to the triplicate wells and incubated at 37°C for another 4 h. Also, 100 μL of color development solution was added and incubated for 10 min to dissolve the crystal completely. Then we measured the absorbance at 572 nm, using a plate reader to calculate the numbers of vital cells in each well.

### Cell migration and cell invasion analysis

*In vitro *cell migration and invasion assays were done as per our previously reported protocols [8,11]. Briefly, 4T1 (control), 4T1-GFP, 4T1-α5, or 4T1-α6.shRNA1 cells (1 × 10^5^) were suspended in 100 μL of serum-free DMEM and added on top of the transwell (8.0 μM pore size; Corning, NY) chambers (for migration) or on top of the Matrigel-coated transwells (30 μL of serum-free DMEM-diluted matrigel; BD Biosciences, San Jose, CA) (for invasion) in triplicate. The DMEM plus 10% FBS culture medium was added to the lower chamber to serve as a chemoattractant. After 6 h (migration) or 24 h (invasion) of incubation at 37°C, the upper surfaces of the filters were carefully wiped with a cotton-tipped applicator. The filters were fixed with 4% paraforrmaldehyde in PBS and stained with 0.1% crystal violet solution. In some cases, cell migration was performed either on fibronectin or laminin matrices. Cells that had migrated or invaded (Matrigel) through the transwell filter pores toward the lower surface of the filters were counted in five different fields under a light microscope.

### Cell cycle analysis

4T1 (control), 4T1-GFP, 4T1-α5, and 4T1-α6-shRNA1 cells were stained for DNA content as per our previously described protocols [12]. Briefly, cells were trypsinized and resuspended in 200 μL ice-cold PBS, after which 4 ml of cold 70% ethanol was added to the mixture. Cells were incubated at 4°C for 45 min. Subsequently, cells were centrifuged at 1,500 g for 10 min at 4°C and resuspended in PI master mix, consisting of propidium iodide 40 ug/ml and RNAse 100 ug/ml (Sigma) at 37°C for 30 min. Cells were then analyzed using an LSR II cytometer. PI staining intensity was determined by Modfit software.

### Cell-cycle genes PCR Array

Total RNA was extracted and measured as previously described [12]. RNA was used to probe GE arrays containing cell-cycle-related genes as per the manufacturer's recommendations (SuperArray Bioscience). Raw data were normalized by GEarray Analyzer software. Data were normalized with control groups (4T1-α5 data normalized with 4T1-GFP and 4T1 α6-1 data normalized with 4T1 control shRNA).

### In-vivo metastasis assay

Twenty five nude mice (NCI) were maintained as per the guidelines of the AALAC and the IACUC LSU Health Sciences Center at New Orleans. These mice were randomly allocated to five groups for tail vein injections of 4T1 control, 4T1-GFP, 4T1-α5, or 4T1-α6-shRNA1 cells. Cells were grown to 80% confluency and harvested in cold PBS. 1 × 10^6 ^cells in 0.1 ml PBS were injected into nude mice via lateral tail vein injection. These mice were then used in the following experiments, each of which was repeated three times.

### Immunohistochemistry

After 4 weeks, mice were euthanized. Their lungs were removed and perfused with buffered formalin through the cannulated trachea. Paraffin wax sections were stained with hematoxylin and eosin (H&E) and Ki67 using standard procedures, and the sections were examined by light microscopy.

### India ink lung staining

At 4 weeks after tail vein injection, mice were sacrificed and India ink (15%) was injected into their lungs through the trachea. The lungs were fixed in Fekete's solution (100 mL of 70% alcohol, 10 mL formalin, and 5 mL glacial acetic acid) at room temperature. After destaining, the lungs were photographed.

### Statistical analysis

Every experiment was repeated at least three times and statistical analysis was performed by one way ANOVA using JMP5.0 Software (SAS Inc, Cary, NC). Fisher's LSD post-hoc test was applied to assess differences of expression levels between each cell type. A value of p ≤ 0.05 was considered statistically significant.

## Results

### In 4T1 cells integrin α5 subunit levels are low and integrin α6 subunit levels are high

**Figure 1:**
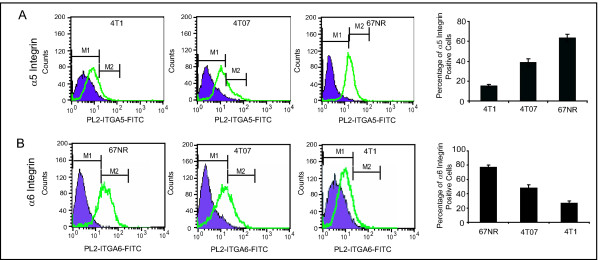
**Flow cytometric analysis of integrins**. Analysis was performed for integrin α5 (**A**) and α6 (**B**) on mouse breast cancer cell lines 4T1 (control), as well as 4T07 and 67NR. The fluorescence signals of unstained cells (primary antibody omitted) for each cell type were presented as blue peaks and served as the basal level of cell-surface expression. The fluorescence signal for integrin α5 and α6 subunits were presented as open peaks. Histograms presented the positive cell percentage of each cell type and the differences are statistically significant (P < 0.01). All experiments were repeated three times.

**Figure 2:**
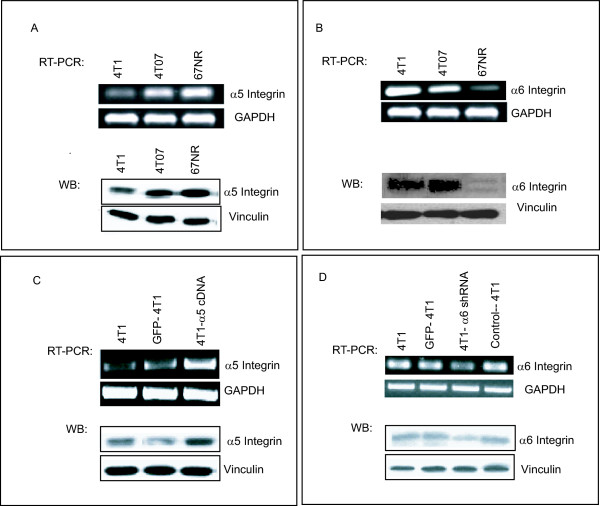
**Validation of expression of integrins by RT-PCR and Western blot analysis**. Expression levels of integrin α5 (**A**) and α6 subunits (**B**) were validated by RT-PCR and Western blotting. RT-PCR was done using the integrin α5- and α6-specific primers. GAPDH was used as the loading control. Cell lysates were prepared and Western blotted with anti-integrin α5 or anti-integrin α6 antibody. Vinculin blotting was used for equal loading. Integrin α5 overexpression (**C**) and α6 knockdown (**D**) were also validated by RT-PCR and Western blot. Stable cell lines overexpressing integrin α5 and knockdown α6 were established. RNA and protein lysates were used to demonstrate the expression of the plasmids.

Since integrins are thought to have a major role in regulating tumor growth and metastasis, we examined the expression levels of integrins in highly invasive (4T1 and 4T07) and noninvasive (67NR) mouse breast cancer cells. Using FACS analysis, we measured the expression levels of 7 integrins, α4, α5, α6, β1, β2, α2β1, and αvβ6. Our results showed that integrin α5 subunit levels were lowest in 4T1, moderate in 4T07, and highest in 67NR cells (Figure 1A). In contrast, integrin α6 subunit expression was highest in 4T1, moderate in 4T07, and lowest in 67NR cells (Additional File 1, Figure 1B). Expression of the other integrins (α4, β1, β2, α2β1, αvβ6) was very low in these cell lines (Additional File 1). RT-PCR and Western blot analysis validated the different levels of expression of integrin subunits α5 and α6 in 4T1, 4T07, and 67NR cells (Figure 2A, B). These findings suggest that the α5 and α6 integrins may have a critical role in tumor growth and metastasis.

To investigate the potential role of integrin subunits α5 and α6 on tumor cell invasion and metastasis of 4T1 cells, we stably transfected these cells with full-length cDNA for mouse integrin α5 subunit and pRNAT-H1.1/neo, which contained the shRNA-control or shRNA-1 for mouse integrin α6 subunit. 4T1-GFP was also used as a control. Mixtures of G418 resistant clones from these stable transfections were tested for the integration of integrin α5 subunit overexpression and integrin α6 subunit knockdown by RT-PCR and Western blot analyses. Our findings demonstrate that the expression levels of integrin α5 in the transfected cells increased as compared with levels in controls (Figure 2C). For integrin α6 subunit knockdown stable cells, both RT-PCR and Western blot analyses demonstrated that shRNA-1 can efficiently target mouse integrin α6 mRNA. Moreover, the expression level of α6 was significantly decreased (Figure 2D). In contrast, shRNA-control (alpha6-2) did not affect integrin α6 subunit expression at either the RNA or protein level (Figure 2D).

### Integrin α5 subunit overexpression and α6 subunit knockdown inhibit 4T1 cell proliferation

**Figure 3:**
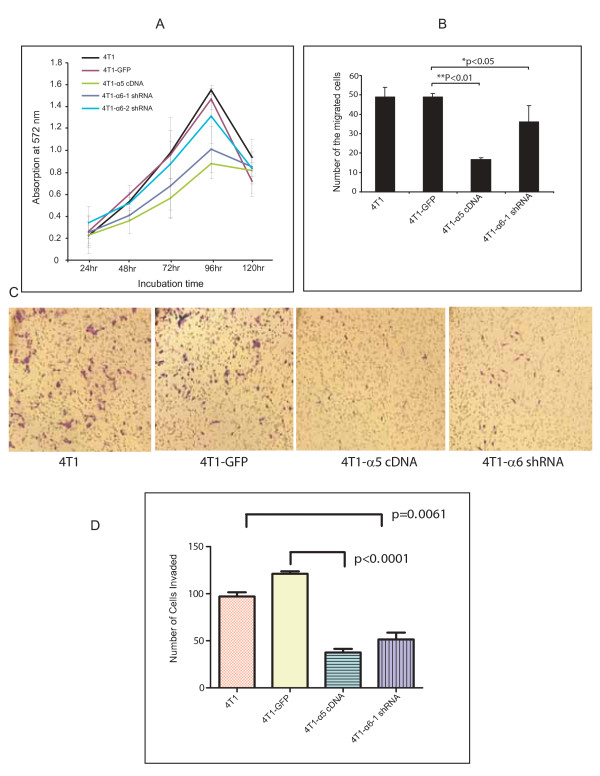
**Modulation of integrin expression inhibits cell proliferation, migration and invasion**. **A**. Cell proliferation of 4T1 cells. 4T1 control, 4T1-GFP, 4T1-α5, and 4T1-α6-shRNA1 cells were seeded and MTT assays done at 24-hr intervals. MTT data were plotted based on the average absorbance value and standard deviation. The Y axis presents the absorbance at 572 nm; the X axis represents incubation time. Black curve: 4T1 control; red curve: 4T1-GFP cells; green curve: 4T1-α5 cells; blue curve: 4T1-α6-shRNA1 cells. ** p < 0.01 (as compared to GFP-4T1). We found that the *in-vitro *proliferation ability of 4T1 cells is inhibited by overexpression of integrin α5 and knockdown of integrin α6. **B. **A transwell migration assay was done for 4T1 (control), GFP-4T1, 4T1-α5, and 4T1-α6-shRNA1 cells. The number of cells that migrated to the lower surface of the filter membrane was counted in 5 random fields under a light microscope (×200). The ability of 4T1 cells to migrate was significantly diminished by overexpression of integrin α5 (p < 0.01) and knockdown of 4T1-α6-shRNA1 (p < 0.05). **C. **Representatives of cell migration figures for each cell type. **D. A **cell invasion assay was done for 4T1, 4T1-GFP, 4T1-α5, and 4T1-α6-shRNA1 cells. 0.1% crystal violet solution was used to stain the lower chamber of transwell. The number of cells that invaded the Matrigel and penetrated the transwell pore to the lower surface was counted in 5 random fields under a light microscope (×200). Overexpression of integrin α5 and knockdown of α6 inhibited the ability of 4T1 cells to invade (p < 0.01).

Since cell proliferation is an important regulator of tumor growth, we examined the potential roles of integrin α5 and α6 genes on cell growth. We used 4T1 control, GFP-4T1, 4T1-α5, and 4T1-α6-shRNA1 cells and measured cell growth by the MTT method. The proliferation curves of 4T1 control and 4T1-GFP cells were quite similar at all time points (Figure 3A). The *in vitro *proliferation ability of 4T1 cells was significantly inhibited at 48 hr by overexpression of integrin α5 subunit (0.30 ± 0.08 versus 0.61 ± 0.07 for 4T1-α5 and 4T1-GFP, respectively; p < 0.01) and 96 hr (0.88 ± 0.14 versus 1.47 ± 0.09 for 4T1-α5 and 4T1-GFP, respectively; p < 0.01). Similarly, integrin α6 subunit knockdown had a significant inhibitory effect on 4T1 cell proliferation at 48 hr (0.41 ± 0.04 versus 0.61 ± 0.07 for 4T1-α6-shRNA1 and 4T1-GFP, respectively; p < 0.01) and 96 hours (1.01 ± 0.21 versus 1.47 ± 0.09, p < 0.01) (Figure 3A). The effect of α6 knockdown on cell proliferation was not quite as robust as that of α5 overexpression. However, alpha6 knockdown did significantly inhibit cell proliferation (Figure 3A). These results suggest that overexpression of α5 and knockdown of α6 reduce cell growth *in vitro*.

### Integrin α5 subunit overexpression and α6 subunit knockdown inhibit 4T1 cell migration and cell invasion

Cell migration, a central process in the development and maintenance of multicellular organisms, has been shown to be involved in tumor formation and metastasis. Therefore, using the classic transwell *in vitro *cell migration assays, we investigated whether integrin α5 subunit overexpression and integrin α6 subunit knockdown affect the migration ability of 4T1 cells. As shown in Figure 3B and 3C, the *in vitro *migration ability of 4T1-α5 cells was significantly decreased as compared to that of 4T1 control and 4T1-GFP cells. The numbers of migrated cells were 17.67 ± 1.0, 49 ± 4.95 and 49 ± 1.86, respectively, for 4T1-α5, 4T1 control, and 4T1-GFP cells (p < 0.01). Similarly, the number of 4T1-α6-shRNA1 cells (36.17 ± 8.25) that migrated through the transwell membrane was significantly lower than the number of 4T1 control cells (49 ± 4.95) and 4T1-GFP cells (49 ± 1.86) that did so (p < 0.05) (Figure 3B, C). Furthermore, to define the functional changes of the integrins, migration of 4T1 and 67NR cells was carried out using either alpha5 integrin or alpha6 integrin functional antibodies. As shown in additional file 2, migration of 67NR cells (that have high levels of α5 integrin) was enhanced in the presence of α5 integrin blocking antibody, further suggesting α5 integrin suppresses cell migration. Also migration of 4T1 cells (that have high levels of α6 integrin) was decreased in the presence of α6 integrin blocking antibody (Additional File 2).

Cell invasion is an important regulator of tumor growth and metastasis. To explore the functional effect of integrin α5 and α6 genes on the invasiveness of 4T1 cells, we did *in vitro *Matrigel invasion assays on these cell lines. Our results showed that the numbers of 4T1-α5 and 4T1-α6-shRNA1 cells that passed through the Matrigel and transwell membrane were significantly lower than the number of cells in the 4T1-GFP control group that did so, indicating that these integrins have important role in cell invasion (Figure 3D).

### Integrin α5 overexpression and α6 knockdown inhibit metastasis

**Figure 4:**
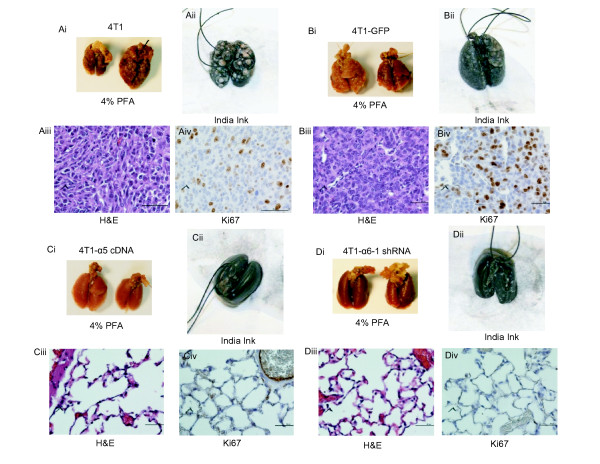
**Overexpression of integrin α5 subunit and knock down of α6 subunit decreased pulmonary metastasis of 4T1 cells**. 1 × 10^6 ^4T1 control, GFP-4T1, 4T1-α5, and 4T1-α6-shRNA1 cells were resuspended in 0.1 ml PBS and used to inject nude mice in their lateral tail veins. After four weeks, the mice were euthanized and their lungs were removed, fixed in 4% paraformaldehyde, and analyzed for metastasis. Representative examples of the lung tissues from each group are shown. **A**: Lungs from 4T1 control-cell-injected mice; **B**: Lungs from 4T1-GFP cells injected mice., **C**: Lungs from 4T1-α5 injected mice., **D**: Lungs from mice injected with 4T1 α6-shRNA1; Ai, Bi, Ci, and Di: Lung images taken right after formaldehyde fixation. Aii, Bii, Cii, and Dii : Lungs stained with India ink:. Aiii, Biii, Ciii, Diii: H&E stained lung sections. Aiv, Biv, Civ, and Div: Ki67 stained lung sections. Scale bar: 100 microns.

Since α5 and α6 have dramatic effects on tumor cell migration and invasion, we hypothesized that these integrins may also have an effect on breast cancer metastasis. Thus we examined the effect of integrins on metastasis in *vivo*. 4T1 cells overexpressing α5 integrin subunit and α6 subunit underexpressing cells (with their controls) were injected into the tail veins of nude mice. Lung metastasis was examined four weeks later. Mice injected with 4T1-α5 and 4T1-α6-shRNA1 cells showed a dramatic decrease in pulmonary metastases, while mice injected with 4T1 control and 4T1-GFP cells produced many metastatic nodules (Figure 4Ai, Bi, Ci, Di). When we dissected the lungs and filled them with 15% India ink, the metastatic tumors, after fixation in Fekete's solution, appeared as white nodules on the black lung surface (Figure 4Aii, Bii, Cii, Dii). H&E staining of the lungs of these mice showed that the presence of α5 and the reduction of α6 reduced lung metastases, while their controls had large numbers of metastases (Figure 4Aiii, Biii, Ciii, Diii). In contrast, the lungs of mice injected with vector control cells were heavily infiltrated with metastases. To our surprise 4T1-α5 and 4T1-α6-shRNA1 cells did not have any effect on breast tumor growth, when we injected them into mammary fat pads (data not shown)., and the reasons for these results are currently unknown. These data suggest that the α5 integrin subunit may function as a metastasis suppressor while α6 subunit may function as a metastasis promoter.

Our *in vitro *studies suggest that integrins have an effect on cell proliferation. To investigate this *in vivo*, we did immunohistochemical analysis for Ki67, a marker for proliferation. In the lungs of mice, altered integrin-expressing tumors had significantly less Ki67 staining than they did in GFP-expressing controls (Figure 4Aiv, Biv, Civ, Div and quantitative data shown in Additional File 3), supporting a role for integrin α5 and α6 subunits in regulating tumor growth, in part through effects on cell proliferation.

**Figure 5:**
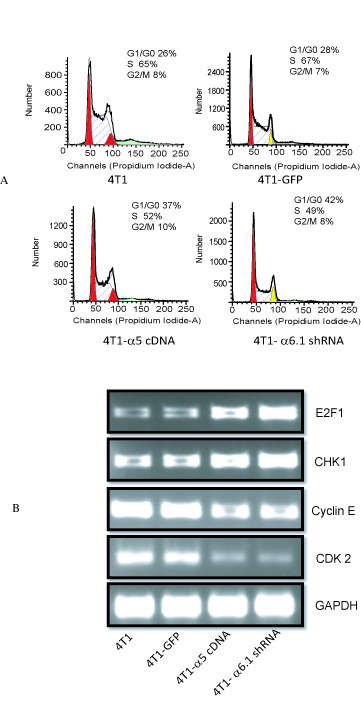
**Modulation of integrins regulates cell cycle progression**. **A: **4T1, GFP-4T1, 4T1-α5, and 4T1-α6-shRNA1 cells were synchronized and stained with propidium iodide for cell-cycle analysis. Cells were arrested at G1/S stage for 4T1-α5 and 4T1-α6-shRNA1 (37 and 42) compared to controls (26 and 28), and have shorter S phase for 4T1α5 and 4T1 α6 shRNA(52, 49) compared to controls (65, 67). No significant changes in G2 phase were noted (4T1 α5:10, 4T1 α6-shRNA1;8, 4T1 control: 8, and 4T1 GFP: 7). B. RT-PCR data. RNA was isolated from all the four cell lines and RT-PCR was performed for E2F1, chk1, Cyclin E and CDK2. GAPDH control was used as a control.

As described, overexpression of integrin α5 and knockdown of integrin α6 subunits inhibited 4T1 cell proliferation ability. Since cell growth is regulated by positive and negative regulators of the cell cycle, we analyzed the effects of alpha5 integrin and alpha6 integrin subunits on cell-cycle progression and its associated proteins. To understand the mechanism whereby integrin α5 and integrin α6 subunits affect cell proliferation, we examined the cell cycle using 4T1 control, 4T1-GFP, 4T1-α5 and 4T1-α6-shRNA1 cells. To determine the effects on cell-cycle progression, we did flow cytometry using propidium iodide. The alpha5 overexpression and knockdown of alpha6 integrin demonstrated a G1/S arrest, which resulted in a decrease in the S-phase population (Figure 5A). This suggested that overexpression of α5 integrin and knockdown of α6 integrin do not allow cells to pass the S phase. As a result, these cells might have inhibited growth.

### Gene expression level of cell-cycle-related genes

To examine the mechanism by which α5 and α6 integrin subunits regulate cell-cycle progression, we did microarray analysis using a cell-cycle gene array containing cell-cycle genes (SA Biosciences). Cell-cycle microarray profiling was done using RNA prepared from 4T1-GFP, 4T1-α5, and 4T1-α6.shRNA1 cells to examine changes in cell-cycle-related genes. We found that the expression of chk-1 and E2F-1 was elevated and cyclin E, CDK2 and cdc25A were decreased in 4T1-α5 and 4T1-α6-shRNA1 cells compared to 4T1-GFP cells (Additional File 4). Later, we validated these data by RT-PCR (Figure 5B and data not shown). Although DST gene levels are significantly high in 4T1-α5 and 4T1-α6-shRNA1 cells, we were unable to validate the data by RT-PCR, and thus it is not further studied. The results suggested that overexpression of alpha5 and knockdown of alpha6 reduces cell growth by enhancing the expression of chk1 and E2F1 and down regulating the expression of cyclin E and CDK2.

### Downregulation of cyclin E/CDK2 complexes

**Figure 6:**
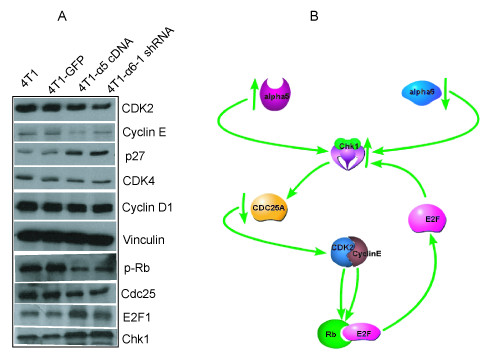
**Modulation of integrin expression regulates expression of cell-cycle modulators known to induce G1 arrest**. A. Western blots show the expression of cyclin E, cdk2, p27, pRb, chk1, cdc25A, and E2F1 in 4T1 control, 4T1 GFP, 4T1 α5 overexpressing, and 4T1 α6 knocked-down cells. **B: **Our current model showing the mechanism of integrin modulation in breast cancer progression. This model shows overexpression of integrin α5 or reduced expression of α6 causes upregulation of chk1. This, in turn, causes downregulation of cdk2/cyclin E to reduce phosphorylation of retinoblastoma protein. This integrin modulation enhances the expression of E2F, which may stimulate chk1 expression, which regulates cdc25a function, in a feedback loop mechanism.

We analyzed the effects of integrin α5 subunit overexpression and α6 subunit knockdown on cell-cycle associated proteins, as shown in Figure 6A, that cells overexpressing α5 integrin and α6 knock-down cells decreased the expression of cyclin E, but not cyclin-D. Consistent with this finding, levels of CDK2, a binding partner of cyclin E, also decreased in the cells. However, no change in the level of CDK4 was observed. These data corroborated the cell cycle array data and suggest that the integrins regulate cell growth through their effects on cell-cycle regulatory proteins. Because it is known that cdc25a regulates CDK2/cyclin E, we examined whether modulation of integrin α5 and α6 subunits have any effect on cdc25a. We found that both overexpression of α5 and knockdown of α6 downregulated the expression of cdc25a (Figure 6A), suggesting that inhibition of cdc25a may be a reason for downregulation of cdk2 and cyclin E.

### Upregulation of Chk1 and P27 expression

We also examined the expression levels of cyclin-dependent kinase inhibitors in the G1/S phase, finding that the expression level of p27 was significantly increased (Figure 6A). Since our cell-cycle array data indicated that α5 overexpression and α6 integrin subunit-reduced expression caused upregulation of chk1 at the RNA level, we examined the expression of chk1 at the protein level. Consistent with the RNA data, protein expression levels were also increased, suggesting that chk1 upregulation could be another mechanism by which these integrins regulate metastasis. Rb phosphorylation is pivotal in controlling cell proliferation by upregulating the G1/S transition of the cell cycle. During the continuous proliferation of cells, pRb is phosphorylated by the activity of G1 phase CDKs such as CDK4 and CDK2, thereby liberating factors that control the S-phase entrance. Consistent with our cyclin E/CDK2 data, these integrin modulations inhibited Rb phosphorylation. Also, to our surprise, we found increased E2F1 levels in these integrin-modulated cells (Figure 6A), which is consistent with the cell-cycle array data. These data suggest α5 and α6 integrin subunits regulate metastasis through their effects on cell cycle regulated proteins.

## Discussion

In this investigation of the expression levels of 8 integrins on the surfaces of cells from mouse breast cancer cell lines 4T1, 4T07, and 67NR, only α5 and α6 integrin subunits showed changes in expression levels in different invasive cell types. The α5 integrin subunit had the lowest expression in highly metastatic 4T1 cells, and little more in 4T07; its highest expression was in 67NR. In contrast, integrin α6 had the opposite expression pattern, as compared to α5 subunit. Our results also showed that overexpression of integrin α5 subunit significantly inhibited 4T1 cell proliferation, cell migration, and cell invasion *in vitro*. Our cell-cycle data indicate that 4T1-α5 cells were arrested at G1/S phase and that a lesser number of cells entered into S phase. Also, our *in vivo *study showed that pulmonary metastasis of 4T1-α5 cells was greatly decreased compared to metastasis of 4T1 and 4T1-GFP cells. Based on these findings, we postulate that integrin α5 subunit functions as an inhibitor of metastasis in mouse breast cancer cell line 4T1. Consistent with our data, lower expression levels of integrin α5 were observed in several cancerous tissues, including hepatocellular carcinoma [7,13], prostate carcinoma [14], and gastric, colorectal, and breast carcinoma [7]. Also previous reports indicated that overexpression of α5β1 inhibits proliferation of human HT29 colon carcinoma cells *in vitro *and reduces the formation of lung colonies and cutaneous metastases *in vivo *[15,16]. Similarly, overexpression of integrin α5 in human hepatocellular carcinoma cells reduces cell proliferation, decreases cell colony formation, and changes the cell-cycle pattern [17]. Elevated levels of α5β1 suppress the transformed phenotype of Chinese hamster ovary cells [18] and reduce tumorigenesis in a mouse model [19]. Niu et al. found that the proliferation of K562 cells could be inhibited by IFNα-2b by restoring the function of integrin α5 and β1 and enhancing their ability to bind to FN, which in turn upregulates FAK gene expression [20].

There are, however, some contradicting reports showing that integrin α5 may function as an oncogene and is associated with malignant tumor progression. Survival analysis of non small-cell lung cancer patients with integrin α5-overexpressing tumors had a worse overall survival rate than did patients whose tumors had normal integrin α5 expression [21]. Further, the integrin α5 molecule has been reported to promote melanoma metastasis *in vitro *and *in vivo *[22]. Integrin α5 up-regulation was identified as the molecular mechanism by which E-cadherin loss promotes tumor progression [23] and ERBB2-mediated transcriptional up-regulation of the α5β1 promotes tumor cell survival under adverse conditions [24]. Thus, integrin α5 has different functions in different carcinoma cell lines. The reasons for these diverse functions of integrin α5 in cancer progression and metastasis are still unclear; however, it has been suggested that α5β1 may affect different signaling cascades and might function differently depending on its adhesion or lack of adhesion to fibronectin [21].

Our results show that knockdown of integrin α6 subunit expression inhibited 4T1 cell proliferation, migration, and invasion. Consistent with these findings, the enhancement of cell migration and invasion by integrin α6 (α6β1 and α6β4) has been shown in pancreatic carcinoma cells [11], and breast cancer [25]. Unlike integrin α5, which functions as either an oncogene or a tumor suppressor, α6 integrin (α6β1 and α6β4) has been implicated only in promoting tumor progression [26-29]. Recent evidence has demonstrated that integrin α6 is necessary for tumorigenicity of a stem-cell-like subpopulation within the MCF7 breast cancer cell line [30]. Integrin α6 can form heterodimers with either β1 or β4. Both heterodimers (α6β1 and α6β4) are receptors for laminin, a component of basement membrane [31]. Higher expression level of α6 (α6β1 and α6β4) was also observed in malignant gastrointestinal stromal tumors [32] and human hepatocellular carcinoma cells [17]. These results suggest that alpha 6 integrin is important in tumorigenesis.

Our results indicate that overexpression of α5 integrin and reduction of α6 integrin subunits prevents cells from passing G1/S phase transition, so that cells in S phase accumulate. Since our work indicated growth arrest in the G1 phase of the cell cycle and based on PCR array data, we investigated whether cyclin E is affected by integrin modulation. Cyclin E/CDK2 complex is a major regulator of G1/S phase transition. The major function of cyclin E is to promote progression through the G1/S phase of the cell cycle by associating with CDK2 to phosphorylate and inactivate retinoblastoma protein and release E2F transcription factors [33]. The activities of the CDKs are regulated by several mechanisms, including binding of cyclins, phosphorylation, and dephosphorylation, as well as binding to CDK inhibitors (CKIs). Overexpression of α5 integrin and downregulation of α6 regulate the expression of cyclin E and CDK2, but not that of cyclin D. P27Kip1, a member of the cip/Kip family of CKIs, alters the activities of cyclin D1 in quiescent cells, leading to failure of the G1/S transition and cell-cycle arrest. Our results indicate that the modulation of the integrins enhances p27 expression. Consistent with this, phosphorylation of pRb is decreased, suggesting that the integrin modulation regulates metastasis affecting Rb phosphorylation. Furthermore, the changes in integrin expression cause upregulation of chk1 and downregulation of Cdc25A expression. Chk1 inhibits Cdc25A expression, whereas Cdc25A phosphatase dephosphorylates and activates CDK2/cyclin E complex during the G1-S transition in proliferating cells[34]. Cdc25A is negatively regulated by phosphorylation of chk 1, which is involved in an S-phase check point pathway that responds to genotoxic stress evoked by DNA damaging agents or irradiation [33]. It is currently unknown how chk1 is induced upon overexpression of α5 or reduction of α6 integrin. In addition, although the mechanism responsible for these changes is not known, our results indicate that E2F1 expression is upregulated as a result of integrin expression changes. Chk1 has been shown to phosphorylate Cdc25A, downregulating its phosphatase activity through distinct mechanisms [34]. For instance, Worms and colleagues reported that chk1 kinase negatively regulates mitotic function of Cdc25A phosphatase through 14-3-3 binding[35].

Based on our results, we propose the following model. Integrin α5 subunit overexpression or reduction of α6 subunit integrin upregulates chk1, which leads to downregulation of cdc25A; this in turn reduces the activity of cyclin E/CDK2. The reduction of activation causes decreased pRb phosphorylation and, as a consequence, cell-cycle growth is inhibited. Simultaneously, p27 is upregulated, which also can downregulate the expression CDK2. Furthermore, E2F is upregulated, which may cause enhanced expression of chk1, a regulator of E2F1. This may upregulate chk1 to downregulate cdc25A in a feedback loop mechanism.

## Competing interests

The authors declare that they have no competing interests.

## Authors' contributions

YW did FACS, cell migration, cell invasion and Western blotting experiments. SS did all animal work and cDNA array experiments. SB did cDNA array experiments, RT PCR and statistical analysis. RR did all cell cycle related Western blots. PJ did specific migration experiments on Firbonectin and Laminin. LB, SH, SD made all the constructs. YW and SKA wrote the manuscript. All authors read and approved the final manuscript.

## Supplementary Material

Additional file 1 **Expression level of integrins of 4T1, 4T07 and 67NR cells by flow cytometry**. Percentage expression values of integrins are shown. Click here for file

Additional file 2 **Effect of blocking antibodies on cell migration**. A transwell migration was done for 67NR cells with IgG control or with alpha5 integrin blocking antibody on fibronectin and 4T1 cells with IgG control or with alpha6 integrin blocking antibody on laminin. Blocking alpha5 integrin enhanced cell migration while blocking alpha6 antibody decreased cell migration and both the effects are statistically significant. Click here for file

Additional file 3 **Quantitation of Ki67 cells in lung sections**. Lung sections stained with Ki67 antibody (described in Aiv, Biv, Civ, and div) were quantitated. The differences between 4T1GFP and 4T1α5, and 4T1GFP and 4T1α6 shRNA1 were statistically significant. Click here for file

Additional file 4 **Cell cycle microarray data**. RNA prepared from 4T1 control cells, 4T1 GFP, 4T1 α5, 4T1 α6-1 were used to screen the expression profile of various cell cycle related genes using cell cycle specific arrays quantitative RT PCR assay. Based on average delta CT values, fold change (regulation) was generated and the values were shown in the table. The candidate genes of interest were depicted in bold. Click here for file
