# Supplementary material for: Integrin subunits alpha5 and alpha6 regulate cell cycle by modulating the chk1 and Rb/E2F pathways to affect breast cancer metastasis
Source: Mol Cancer. 2011 Jul 13;10:84. doi: 10.1186/1476-4598-10-84 (PMC3163626; doi:10.1186/1476-4598-10-84)
Supplement: Additional file 1 — Expression level of integrins of 4T1, 4T07 and 67NR cells by flow cytometry. Percentage expression values of integrins are shown. [file 1476-4598-10-84-S1.DOC]

|  | 4T1 | 4T07 | 67NR |
| --- | --- | --- | --- |
| α4 | 0.06 % | 1.47 % | 0.87 % |
| α5 | 16.1 % | 38.8 % | 62.6 % |
| α6 | 76.9 % | 52.6 % | 26.8 % |
| β1 | 8.05 % | 7.07 % | 1.1 % |
| β2 | 0.13 % | 1.2 % | 0.73 % |
| α2β1 | 8.6 % | 1.74 % | 0.56 % |
| α5β1 | 3.85 % | 27.3 % | 20.2 % |
| αvβ6 | 1.66 % | 3.69 % | 0.08 % |
