# Supplementary figures and images for: Integrin subunits alpha5 and alpha6 regulate cell cycle by modulating the chk1 and Rb/E2F pathways to affect breast cancer metastasis
Source: Mol Cancer. 2011 Jul 13;10:84. doi: 10.1186/1476-4598-10-84 (PMC3163626; doi:10.1186/1476-4598-10-84)

## Slide 1
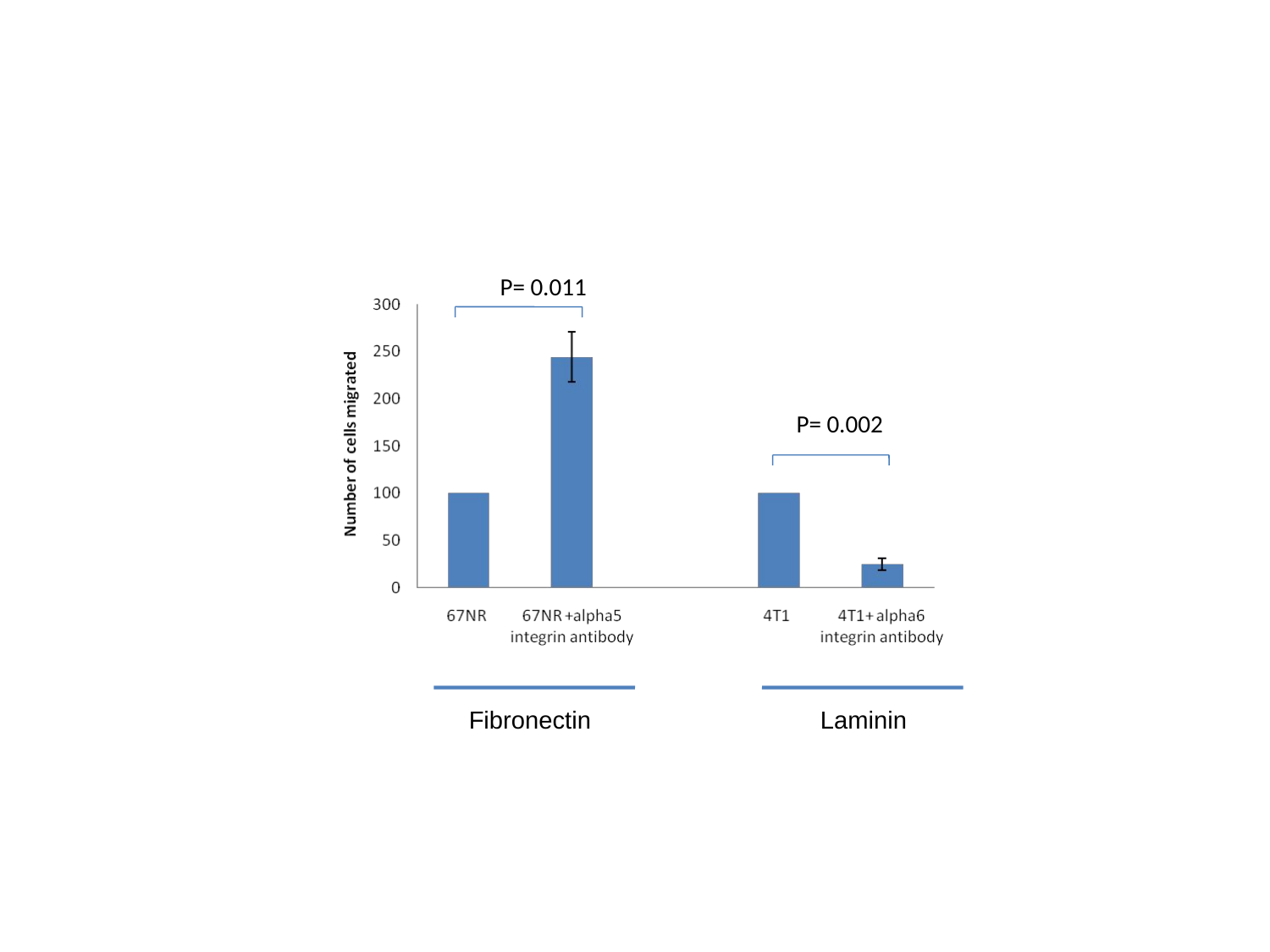

P= 0.011
P= 0.002
Fibronectin		 Laminin

Supplement: Additional file 2 — Effect of blocking antibodies on cell migration. A transwell migration was done for 67NR cells with IgG control or with alpha5 integrin blocking antibody on fibronectin and 4T1 cells with IgG control or with alpha6 integrin blocking antibody on laminin. Blocking alpha5 integrin enhanced cell migration while blocking alpha6 antibody decreased cell migration and both the effects are statistically significant. [file 1476-4598-10-84-S2.PPT]

## Slide 1
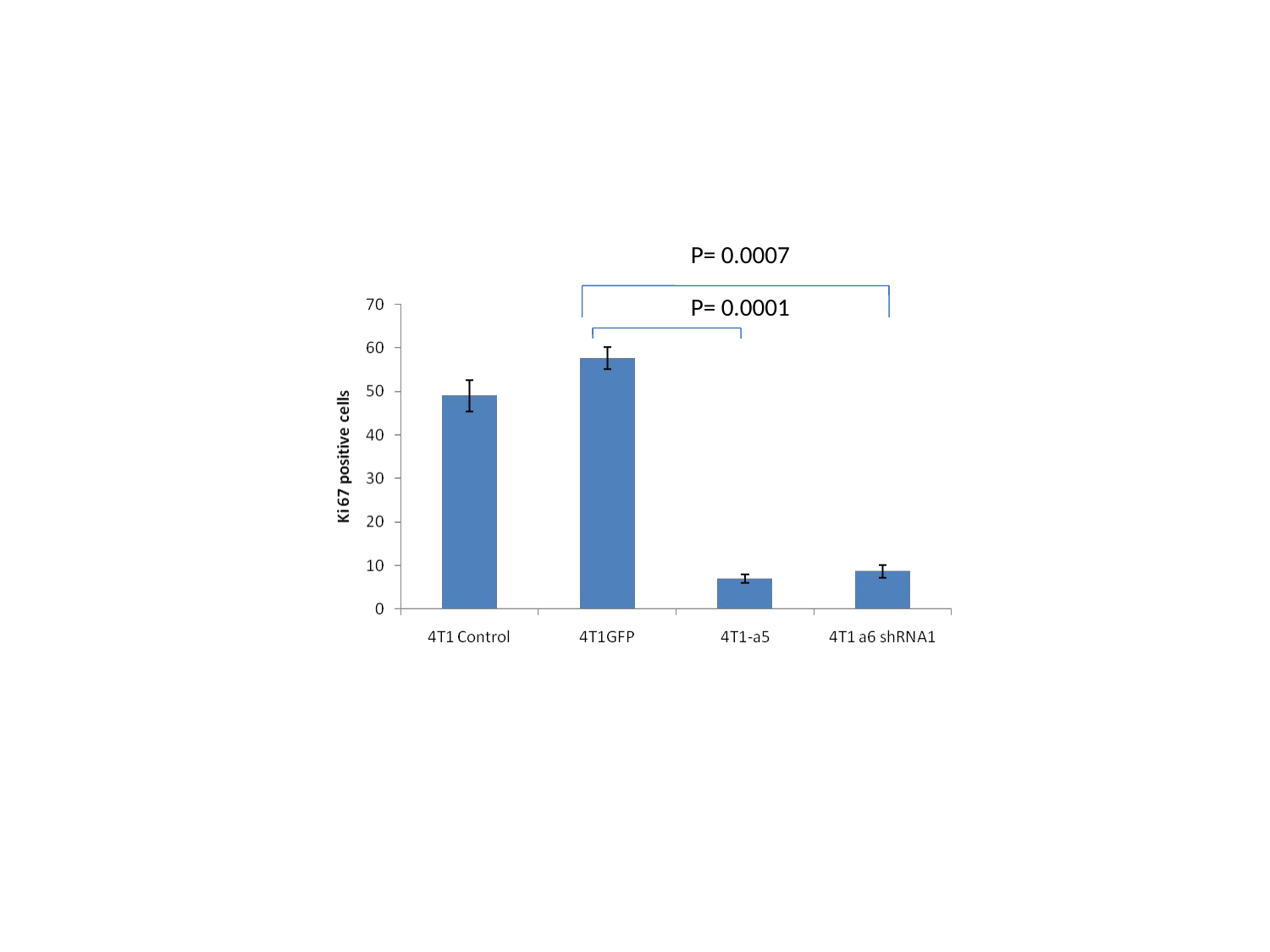

P= 0.0007
P= 0.0001

Supplement: Additional file 3 — Quantitation of Ki67 cells in lung sections. Lung sections stained with Ki67 antibody (described in Aiv, Biv, Civ, and div) were quantitated. The differences between 4T1GFP and 4T1α5, and 4T1GFP and 4T1α6 shRNA1 were statistically significant. [file 1476-4598-10-84-S3.PPT]
