# Supplementary material for: Integrin subunits alpha5 and alpha6 regulate cell cycle by modulating the chk1 and Rb/E2F pathways to affect breast cancer metastasis
Source: Mol Cancer. 2011 Jul 13;10:84. doi: 10.1186/1476-4598-10-84 (PMC3163626; doi:10.1186/1476-4598-10-84)
Supplement: Additional file 4 — Cell cycle microarray data. RNA prepared from 4T1 control cells, 4T1 GFP, 4T1 α5, 4T1 α6-1 were used to screen the expression profile of various cell cycle related genes using cell cycle specific arrays quantitative RT PCR assay. Based on average delta CT values, fold change (regulation) was generated and the values were shown in the table. The candidate genes of interest were depicted in bold. [file 1476-4598-10-84-S4.DOC]

|  |  |  | |  | | |  |
| --- | --- | --- | --- | --- | --- | --- | --- |
| Gene Symbol | Genbank TM | Protein | Up- Down regulation (compared to control group) | | |  | |
|  |  |  | Group 1 | | Group 2 |  | |
| Ccne1 | NM_007633 | Cyclin E1 | **-1.6044** | | **-1.1942** |  | |
| Ccnf | NM_007634 | Cyclin F | 1.9092 | | -1.1376 |  | |
| Cdc25a | NM_007658 | Cell division cycle 25 homolog A (S. pombe) | **-2.184** | | **-2.307** |  | |
| Cdk2 | NM_016756 | Cyclin-dependent kinase 2 | **-1.2075** | | **-1.5063** |  | |
| Cdk4 | NM_009870 | Cyclin-dependent kinase 4 | 1.0091 | | 1.2605 |  | |
| Cdk5rap1 | NM_025876 | CDK5 regulatory subunit associated protein 1 | 1.0446 | | 1.06 |  | |
| Cdkn1a | NM_007669 | Cyclin-dependent kinase inhibitor 1A (P21) | -2.1765 | | -5.4114 |  | |
| Cdkn1b | NM_009875 | Cyclin-dependent kinase inhibitor 1B | 1.9292 | | 2.0477 |  | |
| Cdkn2a | NM_009877 | Cyclin-dependent kinase inhibitor 2A | -1.1034 | | -2.5071 |  | |
| Chek1 | NM_007691 | Checkpoint kinase 1 homolog (S. pombe) | **1.5032** | | **1.5147** |  | |
| Cks1b | NM_016904 | CDC28 protein kinase 1b | 1.0815 | | 1.3004 |  | |
| Ddit3 | NM_007837 | DNA-damage inducible transcript 3 | -1.3727 | | -1.9132 |  | |
| Dnajc2 | NM_009584 | DnaJ (Hsp40) homolog, subfamily C, member 2 | -1.6268 | | -1.9332 |  | |
| Dst | NM_134448 | Dystonin | 10.6516 | | 5.893 |  | |
| E2f1 | NM_007891 | E2F transcription factor 1 | **1.238** | | **1.1802** |  | |
